# Is the Relationship between Body Size and Trophic Niche Position Time-Invariant in a Predatory Fish? First Stable Isotope Evidence

**DOI:** 10.1371/journal.pone.0009120

**Published:** 2010-02-09

**Authors:** Takefumi Nakazawa, Yoichiro Sakai, Chih-hao Hsieh, Tadatoshi Koitabashi, Ichiro Tayasu, Norio Yamamura, Noboru Okuda

**Affiliations:** 1 Center for Ecological Research, Kyoto University, Otsu, Japan; 2 Institute of Oceanography, National Taiwan University, Taipei, Taiwan; 3 Institute of Ecology and Evolutionary Biology, National Taiwan University, Taipei, Taiwan; 4 Research Institute for Humanity and Nature, Kyoto, Japan; University of Liverpool, United Kingdom

## Abstract

Characterizing relationships between individual body size and trophic niche position is essential for understanding how population and food-web dynamics are mediated by size-dependent trophic interactions. However, whether (and how) intraspecific size-trophic relationships (i.e., trophic ontogeny pattern at the population level) vary with time remains poorly understood. Using archival specimens of a freshwater predatory fish *Gymnogobius isaza* (Tanaka 1916) from Lake Biwa, Japan, we assembled a long-term (>40 years) time-series of the size-dependence of trophic niche position by examining nitrogen stable isotope ratios (*δ* ^15^N) of the fish specimens. The size-dependence of trophic niche position was defined as the slope of the relationship between *δ* ^15^N and log body size. Our analyses showed that the slope was significantly positive in about 60% of years and null in other years, changing through time. This is the first quantitative (i.e., stable isotope) evidence of long-term variability in the size-trophic relationship in a predatory fish. This finding had implications for the fish trophic dynamics, despite that about 60% of the yearly values were not statistically different from the long-term average. We proposed hypotheses for the underlying mechanism of the time-varying size-trophic relationship.

## Introduction

Body size exerts a critical influence on ecological processes, such as predation and predation avoidance, which in turn regulate intra- and interspecific interactions, population dynamics, and thus community structure within food webs [1]–[3]. Because trophic interactions are often size-dependent, understanding size structure within a population is of fundamental importance. Variation in individual body size is commonly observed within populations [4]. Many species undergo considerable increases in body size during their development, during which they use different resources, resulting in ontogenetic niche shift [5], [6]. Ontogenetic niche shift can alter trophic relationships among specie, because one individual has distinct size classes or stages through its development, which play different trophic roles in food-webs [7]. In theory, such effects play an important role in affecting food-web dynamics [8], [9]. Characterizing the relationship between individual body size and trophic niche position is thus needed if we are to understand and ultimately predict how population and/or food-web dynamics are mediated by size-dependent trophic interactions.

A fundamental, but unresolved, question in the study of size-trophic relationship is whether the size-dependence of trophic niche position is time-invariant or not within species and how it varies with time. The importance of this question is obvious in the study of food-web dynamics, as abovementioned. Further, the temporal variability of size-trophic relationships is of particular importance for understanding energy flow in ecosystem-based fishery management. Trophic dynamics of populations, a major concern in ecosystem studies [10], have traditionally been estimated from temporal changes in size structure based on a given size-trophic relationship [11], [12] (see also [13] for community-level studies). These studies are based on the largely untested assumption that the size-dependence of trophic niche position is time-invariant. A recent study by Jennings et al. [14] suggested that the size-dependence of trophic level is constant for several marine fish species. However, their three-year data may not have been sufficiently long to conclude that the size-dependence of trophic level is time-invariant. Long-term time-series data are therefore needed to capture the dynamics of size-trophic relationships and to directly test the temporal variability in the relationship between body size and trophic niche position.

In the present study, we address the question of whether the size-dependence of trophic level changes through time, using a predatory fish. In fish species, trophic level is generally a good indictor of trophic niche position and its size-dependency represents the trophic ontogeny pattern at the population level [13]. Through direct observation of stomach contents or foraging behaviors, it may have been suggested that size-trophic relationship is not constant (e.g., due to size-related optimal foraging [15]). However, quantitative (i.e., stable isotope) evidence is lacking. Recent advances in stable isotope ecology represent a significant contribution to trophic level estimation. In particular, the use of stable isotopes in aquatic systems has suggested that strong positive size-trophic relationships exist within fish populations (e.g., juveniles as planktivorous and adults as piscivorous; see [13], [16] for reviews), albeit the information is still a “snapshot” on a time scale of population dynamics.

In the present study, to improve our understanding of the long-term variability (and potentially the regulation) of the intraspecific size-trophic relationship, we investigated size-based variation of the nitrogen stable isotope ratios (*δ* ^15^N) of archival specimens of a freshwater predatory fish *Gymnogobius isaza* (Tanaka 1916). We constructed a >40 year time-series of the size-dependence of trophic level. In addition, we analyzed the data to show the implications for the fish trophic dynamics. Finally, we discussed hypotheses for determinants of the size-trophic relationships.

## Materials and Methods

### Fish Species and Its Feeding Habits

*G*. *isaza* is a freshwater goby endemic to Lake Biwa, Japan. Whereas most gobiid fish are benthic, *G*. *isaza* has adapted to a pelagic habitat with its strong swimming ability. *G*. *isaza* migrates from the pelagic to the littoral zone for breeding in spring. The hatched larvae disperse offshore to grow from summer to winter, reaching maturity in the next spring [17]. They are usually annual and die after spawning, with some fish surviving to the second year. This species is omnivorous, feeding on algae, detritus, zooplankton (mainly Cladocera and Copepoda), profundal gammarids, juvenile fish and shrimps [18]. In order to reconstruct the past pelagic environments, Ogawa et al. [19] examined the *δ* ^15^N of fish specimens. They found enrichment in *δ* ^15^N of the fish specimens in the late 1960s, and this enrichment was synchronized with that of the sediment core (a proxy to pelagic primary producers). Thus, they argued that the fish trophic level was invariant with time. However, the size-dependence of trophic level was not considered in their study, because they analyzed only one or a few specimens per year.

### Sampling

Fish were collected annually between 1962 and 2004 using a trawl net. Fish specimens were not collected in 1991, 1992, and 2004 because the population density was too low [20]. The specimens were initially fixed in 10% formalin and subsequently preserved in 70% ethanol (see [19] for details). For the stable isotope analysis, we selected 20 specimens per year from autumn to winter (mainly December) collections in a way that represented a nearly uniform coverage of the body size range of each sampling year (see Table S1 for their maximum and minimum body size). Here we used total body length (but not body mass) as our measure of body size, as body length was strongly correlated with wet weight for the entire data set (*n* = 800, *r* ^2^ = 0.96, *p*<0.0001).

### Stable Isotope Analysis

A small piece of muscle tissue was excised from the dorsal part of the lateral body of each specimen. After desiccation at 60^o^C for at least two days and pulverization, the tissue samples were folded into tin capsules, and *δ* ^15^N was measured using a mass spectrometer (Finnigan MAT delta S, Germany). The analytical precision was ±0.2‰. The natural abundance of *δ* ^15^N was expressed in per mil (‰) deviation from the standard (atmospheric N_2_), as follows;
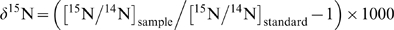



Although long-term preservation in organic solvents such as formalin and ethanol may alter the tissue isotopic signature [21], preservation effects on tissue *δ* ^15^N are generally very small and stable over six months [22]. In addition, relative variation of isotopic values within an annual group of specimens would have experienced the same potential isotopic alteration; therefore, we consider that isotopic denaturation due to long-term preservation is not a critical issue in our analysis of size-dependent trophic level within each year.

### Data Analysis

The size-dependence of trophic level was defined as the slope of the regression *δ* ^15^N versus log_10_ total body length for each sampling year [13], [16]:
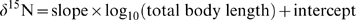



The significance of the slope for each year is tested using bootstrapped regression with accelerated bias correction [23]. To avoid the statistical problem associated with multiple comparisons, we used Bonferrini correction with α = 0.00125 (0.05/40) to determine the confidence limit for hypothesis tests. Note that we do not refer to absolute values of fish trophic level in the present study, because we focus on size-dependence of trophic level in a relative sense. Although *G*. *isaza* has a variety of food items (see above), their basal food is phytoplankton because this species inhabits pelagic waters and their main diet, profundal zoobenthos and zooplankton, show a strong reliance on phytoplankton in Lake Biwa [24]. Thus, our estimation of the slope using only fish *δ* ^15^N will be justified; that is, even though the *δ* ^15^N signature of the baseline has changed over time, it is unlikely to affect our results (but see [25] for interspecific size-trophic relationships). It should be also noted that in fish, *δ* ^15^N fractionation is independent of body size [26], so that the regression slope can reflect size-dependence of trophic level.

We then use the time series of the slopes to exhibit the temporal variation of size-dependence of trophic level. To further confirm the existence of significant differences in slope among years, we implemented a general mixed model as follows:
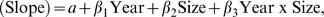



where Size is log_10_ (total body length). If *β_3_* is significantly different from zero, one can confirm that slopes differ significantly among years.

**Figure 1:**
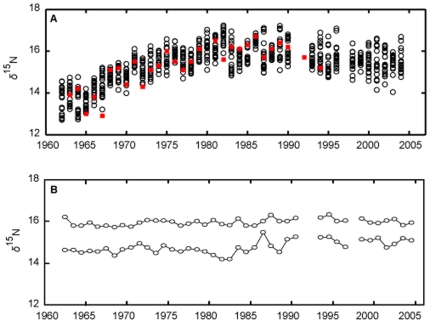
Measured and simulated time-series of *δ* ^15^N. (a) Open circle and red squares represent individual values measured in the present study and Ogawa et al [19], respectively. (b) Two lines represent the maximum and minimum values of *δ* ^15^N, respectively, calculated from the maximum or minimum values of total body length of each sampling year using the time-averaged size-trophic relationship (the blue line in Figure 2).

The *δ* ^15^N of *G*. *isaza* changed over the 40 years (Figure 1a; see also Table 1; see Table S1 for the maximum and minimum values of *δ* ^15^N). It increased by about 3 ‰ during the 1960s and 1970s at the population level, and thereafter it decreased slightly or remained constant. This trend was basically similar to that reported by Ogawa et al. [19]. In the present study, to illustrate the implications of the temporal variability of size-trophic relationship for the fish trophic dynamics, we conducted simulations in the following steps. First, we obtained the long-term average relationship between body size and *δ* ^15^N using data from all individuals. Next, assuming that this relationship between body size and *δ* ^15^N was time-invariant, we calculated the maximum and minimum *δ* ^15^N (i.e., trophic niche width) of the fish population from the maximum and minimum body size of each sampling year. This simulation produced the predicted *δ* ^15^N dynamics that do not include the temporal variability of the size-trophic relationship; by contrast, the measured *δ* ^15^N dynamics is a result encompassing the temporal variability. Then, we compared the measured and predicted *δ* ^15^N dynamics.

**Table 1:** Result of general mixed model.

Effect	DF	error DF	F Value	Pr > F
Year	39	720	9.36	<.0001
Size	1	720	531.97	<.0001
Size x Year	39	720	8.73	<.0001
Size is log_10_ (total body length).				

### Evaluation of Potential Sampling Bias

One might concern that bias in estimating the slopes may arise due to sampling procedures. For example, the dispersion in fish size may be high or low in some years, which may produce biased high or low slope values. To investigate this issue, we used body size range (i.e. log_10_ (maximum total body length) - log_10_ (minimum total body length)) as a reasonable measure of dispersion, because distribution of size data within any year is largely uniform within the body size range, owing to our sampling design. In addition, the annual variations in maximum and minimum body sizes were consistent with those in the previous work [27] which covered data from 1975 to 2002 (maximum body length: *n* = 24, *r* ^2^ = 0.23, *p*<0.05; minimum body length: *n* = 23, *r* ^2^ = 0.44, *p*<0.001). A regression of the slopes versus body size ranges was not significant (*n* = 25, *r* ^2^ = 0.12, *p* = 0.10), thereby indicating that sampling bias effect on the slope values is minimal.

## Results and Discussion

**Figure 2:**
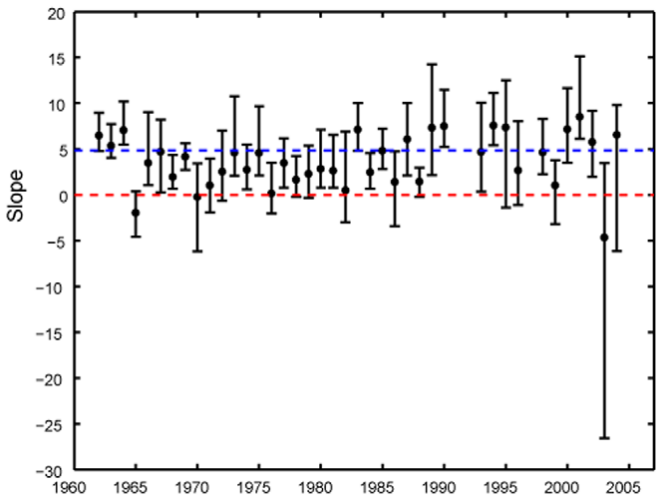
The slope of the relationship between *δ* ^15^N and body size for each sampling years. The vertical bars represent 99.875% bootstrapped confidence limits based on accelerated bias correction (see text). If the vertical bars include zero (the red line), the slope is not significant. The blue line indicates the long-term average slope calculated from all data pooled.

We found that the within-year variation of *δ* ^15^N was up to about 2 ‰ (i.e., 0.6 trophic step; Figure 1a). The relationship between body size and trophic level was significantly positive in about 60% years (25 out of 40 years), while it was not significant in other years (Figure 2; see Table S1 for *r* ^2^ of the annual regression slope). The maximum value of the statistically significant slope was 8.52 in 2001. Results of general mixed model analysis showed that the year, body size, and year-body size interaction effects were significant (Table 1), indicating that the relationships between *δ* ^15^N and body size varied through time. That is, the slope (i.e., the size-dependence of tropic level) was not time-invariant; it varied over the course of 40 years, contrary to a conventional view. To our knowledge, this is the first stable isotope evidence of long-term variation in intraspecific relationships between body size and trophic niche position in a predatory fish.

Although 60% of the yearly slope values were not statistically different from the long-term average (25 out of 40 years; Figure 2), our finding had important implications for the estimation of fish trophic dynamics; that is, assuming a constant size-trophic level relationship and using body size to infer trophic dynamics for a population is not always justified. The essence of this argument is provided by statistically predicting trophic (*δ* ^15^N) dynamics of *G*. *isaza* from body size of each sampling year (Figure 1b), using the long-term average slope (blue line in Figure 2). These calculations showed that the variability of the trophic dynamics would be masked if the slope was assumed to be time-invariant (compare Figures 1a and 1b). Notably, the increasing trend of *δ* ^15^N in the 1960s and 1970s in Figure 1a disappeared (Figure 1b). Our simulation exercise points out that if one wishes to estimate trophic dynamics by using only size information under the assumption that the size-trophic relationship is time-invariant, one must confirm first that the possible determinants of the size-trophic relationships is also time-invariant (see below). Such a problem has not been fully considered in the previous studies of size-based food-web analysis, [11], [12].

What is needed next is to investigate the mechanism underlying the observed long-term variations in the slopes (Figure 2). Here, we propose potential explanations for our observations, although we cannot fully explain the pattern with the data currently at hands. First, we consider that the maximum fish body size may have affected the slope [28]. In aquatic ecosystems, larger fish individuals generally have a higher trophic level because they can effectively consume larger prey items that have higher trophic levels [13], [16]. The point here is that fish increases body size (via growth) gradually, whereas the trophic level of their prey in diet rises stepwise (e.g. from copepods to shrimps) along with the gradual increase in fish body size, because diet items of predatory fish are generally gape-limited [29]-[31]. That is, fish can consume larger prey items when they reach a body size threshold. As large fish (above a certain threshold) will have a higher trophic level, the presence of such large individuals significantly determines the positive relationship between body size and trophic level within the population [28]. If this mechanism is at work for the fish we studied here, it is expected that an increase in the maximum body size would have caused an expansion of potential food items, allowing larger fish to consume larger prey items (e.g. shrimp and juvenile fish) with much higher *δ* ^15^N than smaller prey (e.g. zooplankton and profundal zoobenthos). Indeed, the slope dynamics was apparently synchronized with the fish body size dynamics reported in the previous work (see Figure 3 in [21]). This scenario is statistically supported by the positive correlation between the slopes and the maximum body sizes (*p*<0.05; Figure S1). However, this is just a correlation but not direct evidence. Future considerations of the stomach contents and the *δ* ^15^N of potential food items are still needed for a definitive conclusion, which will be tested in our future work.

On the other hand, we found that the relationship between the slopes and the minimum body sizes was also significantly positive (*p*<0.01; Figure S1), which implies that size-dependence of trophic level becomes less clear in the presence of very small individuals. One possible ecological explanation is that, if small fish (below a certain threshold) cannot eat larger preys and thus they have a narrow range of food items (i.e., no size-dependence within smaller size classes), then the slopes may decrease with decreasing minimum body size of the fish population. However, it can also be argued that the positive correlation between the slope and the minimum body size may be the byproduct of the positive relationship between the maximum and minimum body sizes (*n* = 40, *r* ^2^ = 0.18, *p*<0.01). As indicated above, more direct evidence is therefore needed to better identify the underlying mechanisms of the time-varying slopes.

We consider that prey availability and competition intensity might also affect the within-population variation in resource acquisition [32], [33] and possibly the size-dependence of trophic level. Considering that prey preference or capture success of the fish is size-dependent (see above), it is reasonable to assume that larger fish have the ability to catch a wide size range of prey but prefer larger one (with a high trophic level), while smaller fish have to use only smaller preys. Under this condition, larger fish may have to shift to smaller items if competition is strong within the large size class [32]. Therefore, we expect that the slope will increase with increases in larger prey availability (relative to smaller one) to larger fish individuals. This scenario, however, cannot be easily evaluated here, because at present we do not have enough data on temporal changes in the body size distribution of the fish and the biomass of the potential prey items. In addition, other factors may be responsible for the time-varying slopes. For example, some prey species may have changed their trophic levels through time for some reasons. This will readily affect the size-trophic relationship of the fish, even without any changes in the fish diets. This possibility can be also tested in our future work investigating temporal changes in the fish stomach contents and the prey *δ* ^15^N.

In conclusion, using long-term stable isotope data, for the first time we have demonstrated that size-dependence of trophic level is not time-invariant in a predatory fish. Our study used specimens of a single species to test the temporal variability, but we consider that this phenomenon will be widespread in nature, especially where population size-structure or prey availability fluctuates significantly with time. If so, it is illuminating and also quite important to investigate how temporal variability in size-dependence of trophic interactions would affects food-web dynamics, because time-invariant size-dependence has been conventionally employed as a key assumption in modeling trophic interactions in size-structured food-web models [2], [3] (but see [8]). We suggest that further studies should identify and quantify key factors affecting the size-dependence of trophic niche position, in order to effectively link individual-level foraging behaviors to size-structured food-web dynamics.

## Supporting Information

Figure S1 Positive correlations of the slopes with the maximum and minimum total body lengths of G. isaza were found. Note here that we used only the statistically significant slopes for the analysis (as shown in Figure 1). The insignificant slope in some years may be attributed to a body size range that is too small to detect a clear correlation statistically. As such, these slope values cannot be realizably estimated and was, therefore, eliminated for this analysis.
(0.08 MB DOC)

Table S1 The maximum and minimum values of body size and δ15N of analyzed specimens and r2 of the regression of δ15N versus body size for each sampling year
(0.12 MB DOC)
