# Supplementary figures and images for: Is the Relationship between Body Size and Trophic Niche Position Time-Invariant in a Predatory Fish? First Stable Isotope Evidence
Source: PLoS One. 2010 Feb 9;5(2):e9120. doi: 10.1371/journal.pone.0009120 (PMC2817743; doi:10.1371/journal.pone.0009120)

**Figure S1** The relationship of the slopes with the maximum and minimum body sizes


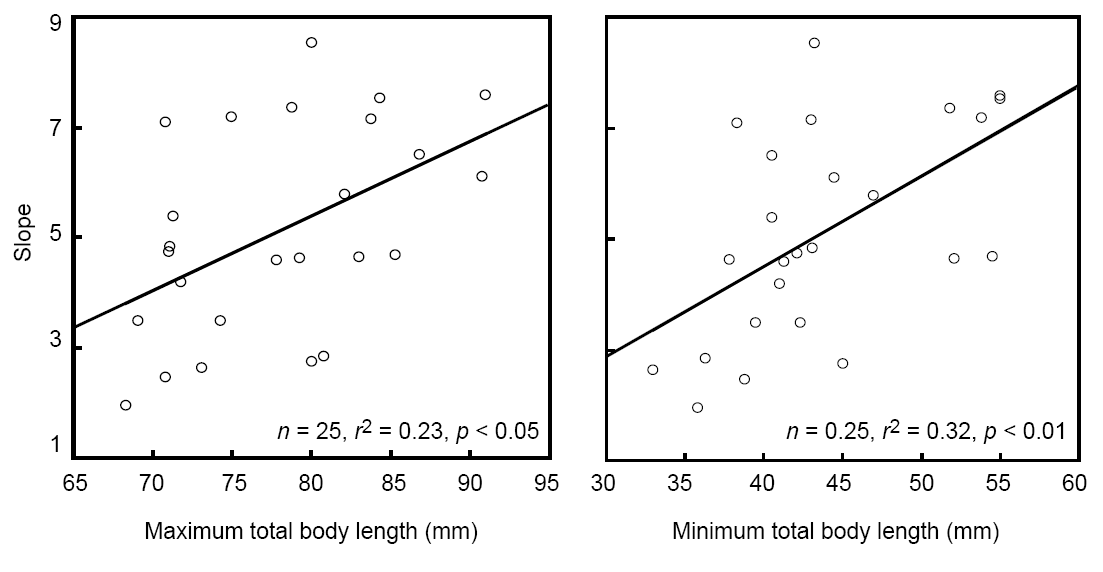

Supplement: Figure S1 — Positive correlations of the slopes with the maximum and minimum total body lengths of G. isaza were found. Note here that we used only the statistically significant slopes for the analysis (as shown in Figure 1). The insignificant slope in some years may be attributed to a body size range that is too small to detect a clear correlation statistically. As such, these slope values cannot be realizably estimated and was, therefore, eliminated for this analysis. (0.08 MB DOC) [file pone.0009120.s001.doc]
