# Supplementary material for: Is the Relationship between Body Size and Trophic Niche Position Time-Invariant in a Predatory Fish? First Stable Isotope Evidence
Source: PLoS One. 2010 Feb 9;5(2):e9120. doi: 10.1371/journal.pone.0009120 (PMC2817743; doi:10.1371/journal.pone.0009120)
Supplement: Table S1 — The maximum and minimum values of body size and δ15N of analyzed specimens and r2 of the regression of δ15N versus body size for each sampling year (0.12 MB DOC) [file pone.0009120.s002.doc]

**Table S1** The maximum and minimum values of body size and *δ*^15^N of analyzed specimens and *r*^2^ of the regression of *δ*^15^N versus body size for each sampling year

| Year | Date | Sample size | Total body length (mm) | | Wet weight (g) | | *δ*^15^N (‰) | | *r*^2^ |
| --- | --- | --- | --- | --- | --- | --- | --- | --- | --- |
|  |  |  | Max. | Min. | Max. | Min. | Max. | Min. |  |
| 1962 | 9-Dec | 20 | 86.8 | 40.5 | 5.8 | 0.5 | 14.56 | 12.72 | 0.89 |
| 1963 | 27-Nov | 20 | 71.3 | 40.5 | 3.0 | 0.4 | 14.73 | 13.23 | 0.90 |
| 1964 | 25-Dec | 20 | 70.8 | 38.3 | 2.9 | 0.3 | 14.77 | 12.74 | 0.88 |
| 1965 | 10-Dec | 20 | 76.0 | 39.8 | 2.6 | 0.2 | 14.20 | 13.04 | 0.30 |
| 1966 | 10-Nov | 20 | 69.1 | 39.5 | 2.7 | 0.4 | 14.71 | 13.40 | 0.51 |
| 1967 | 6-Dec | 20 | 71.0 | 42.1 | 3.9 | 0.5 | 15.22 | 13.22 | 0.46 |
| 1968 | 10-Dec | 20 | 68.3 | 35.8 | 2.8 | 0.3 | 15.23 | 14.36 | 0.46 |
| 1969 | 5-Dec | 20 | 71.8 | 41.0 | 3.9 | 0.5 | 15.44 | 14.15 | 0.86 |
| 1970 | 10-Dec | 20 | 69.1 | 43.1 | 3.0 | 0.6 | 15.39 | 13.99 | 0.00 |
| 1971 | 10-Dec | 20 | 76.0 | 47.1 | 4.5 | 0.8 | 16.11 | 14.99 | 0.09 |
| 1972 | 8-Dec | 20 | 80.9 | 43.1 | 4.9 | 0.6 | 15.66 | 14.41 | 0.36 |
| 1973 | 10-Dec | 20 | 79.3 | 37.8 | 4.9 | 0.4 | 16.24 | 14.53 | 0.53 |
| 1974 | 22-Dec | 20 | 80.0 | 45.0 | 4.9 | 0.5 | 16.04 | 14.90 | 0.51 |
| 1975 | 10-Dec | 20 | 77.8 | 41.3 | 4.2 | 0.6 | 16.58 | 14.85 | 0.53 |
| 1976 | 10-Nov | 20 | 71.0 | 39.3 | 3.3 | 0.3 | 16.48 | 15.45 | 0.00 |
| 1977 | 10-Dec | 20 | 74.3 | 42.3 | 3.2 | 0.5 | 16.15 | 14.69 | 0.49 |
| 1978 | 13-Dec | 20 | 78.8 | 41.3 | 3.6 | 0.5 | 16.08 | 14.93 | 0.23 |
| 1979 | 9-Nov | 20 | 73.0 | 39.5 | 3.7 | 0.4 | 16.59 | 15.48 | 0.30 |
| 1980 | 10-Nov | 20 | 80.8 | 36.3 | 4.3 | 0.3 | 17.03 | 15.11 | 0.44 |
| 1981 | 10-Dec | 20 | 73.1 | 33.0 | 3.6 | 0.3 | 16.79 | 15.42 | 0.43 |
| 1982 | 10-Dec | 20 | 71.5 | 33.1 | 3.4 | 0.2 | 17.23 | 15.90 | 0.01 |
| 1983 | 10-Dec | 20 | 83.8 | 43.0 | 5.7 | 0.6 | 16.95 | 14.83 | 0.84 |
| 1984 | 10-Dec | 20 | 70.8 | 38.8 | 3.5 | 0.4 | 16.09 | 15.05 | 0.50 |
| 1985 | 10-Dec | 20 | 71.1 | 43.1 | 3.6 | 0.5 | 16.50 | 15.39 | 0.77 |
| 1986 | 10-Dec | 20 | 78.5 | 61.0 | 4.1 | 2.1 | 16.86 | 16.10 | 0.05 |
| 1987 | 9-Dec | 20 | 90.8 | 44.5 | 7.3 | 0.6 | 17.12 | 14.97 | 0.67 |
| 1988 | 10-Dec | 20 | 78.5 | 38.8 | 4.9 | 0.4 | 16.29 | 15.57 | 0.35 |
| 1989 | 8-Dec | 20 | 78.8 | 51.8 | 4.6 | 1.0 | 17.22 | 15.10 | 0.56 |
| 1990 | 13-Dec | 20 | 84.3 | 55.0 | 6.6 | 1.5 | 16.81 | 15.28 | 0.78 |
| 1991 | - | - | - | - | - | - | - | - | - |
| 1992 | - | - | - | - | - | - | - | - | - |
| 1993 | 25-Dec | 20 | 85.3 | 54.5 | 6.7 | 1.4 | 16.27 | 15.12 | 0.60 |
| 1994 | 25-Dec | 20 | 91.0 | 55.0 | 7.5 | 1.2 | 16.89 | 14.97 | 0.82 |
| 1995 | 10-Oct | 20 | 78.8 | 49.0 | 5.8 | 0.8 | 16.70 | 14.76 | 0.58 |
| 1996 | 20-Oct | 20 | 79.3 | 43.8 | 5.2 | 0.6 | 17.08 | 15.03 | 0.20 |
| 1997 | - | - | - | - | - | - | - | - | - |
| 1998 | 9-Dec | 20 | 83.0 | 52.1 | 7.0 | 1.1 | 16.37 | 14.63 | 0.63 |
| 1999 | 15-Dec | 20 | 75.8 | 50.8 | 4.6 | 1.1 | 15.93 | 15.02 | 0.07 |
| 2000 | 13-Dec | 20 | 75.0 | 53.8 | 5.2 | 1.3 | 16.41 | 14.58 | 0.68 |
| 2001 | 15-Dec | 20 | 80.0 | 43.2 | 5.1 | 0.7 | 16.61 | 14.15 | 0.77 |
| 2002 | 12-Dec | 20 | 82.1 | 47.0 | 6.8 | 0.4 | 16.29 | 14.42 | 0.64 |
| 2003 | 5-Dec | 20 | 71.8 | 53.1 | 3.7 | 1.3 | 15.95 | 14.04 | 0.13 |
| 2004 | 30-Dec | 20 | 76.1 | 50.5 | 4.7 | 1.1 | 16.69 | 14.76 | 0.54 |
